# Castleman's Disease and Posttransplant Lymphoproliferative Disorder after Liver Transplant: 3-Year Follow-Up

**DOI:** 10.1155/2018/9324872

**Published:** 2018-01-28

**Authors:** Lokesh K. Jha, Laura L. Ulmer, Marco A. Olivera-Martinez, Timothy M. McCashland, Kai Fu, Fedja A. Rochling

**Affiliations:** ^1^Division of Gastroenterology & Hepatology, Department of Internal Medicine, University of Nebraska Medical Center, Omaha, NE, USA; ^2^Department of Hematology/Oncology, University of Nebraska Medical Center, Omaha, NE, USA

## Abstract

A 59-year-old male with a history of hepatitis C cirrhosis and history of hepatitis B exposure presented 8 months after orthotopic liver transplant (LT) with fever, fatigue, myalgia, night sweats, nonproductive cough, and shortness of breath. Bone marrow biopsy for pancytopenia was positive for Epstein-Barr virus (EBV) DNA. Lymph node biopsy for lymphadenopathy on imaging showed human herpes virus 8 (HHV8) associated Castleman's disease. Treatment included valganciclovir, rituximab, and prednisone taper with eventual discontinuation. Quantitative HHV8 DNA was initially 611,000 DNA copies/mL and was later undetectable at 6 months following treatment and remained undetectable at 3-year follow-up.

## 1. Introduction

Castleman's disease is a lymphoproliferative disorder associated in some cases with the human immunodeficiency virus (HIV) and human herpes virus 8 (HHV8). It can be unicentric or multicentric. HHV8 related clinical manifestations range from Kaposi Sarcoma (KS), intracavity primary effusion lymphoma (PEL) and systemic multicentric Castleman disease (MCD). HHV8 is more prevalent in sub-Saharan African, Latin American, Caribbean, Mediterranean, and Middle Eastern countries. This is commonly a donor derived disorder and may in fact involve the transplanted organ [1].

## 2. Case

**Figure 1:**
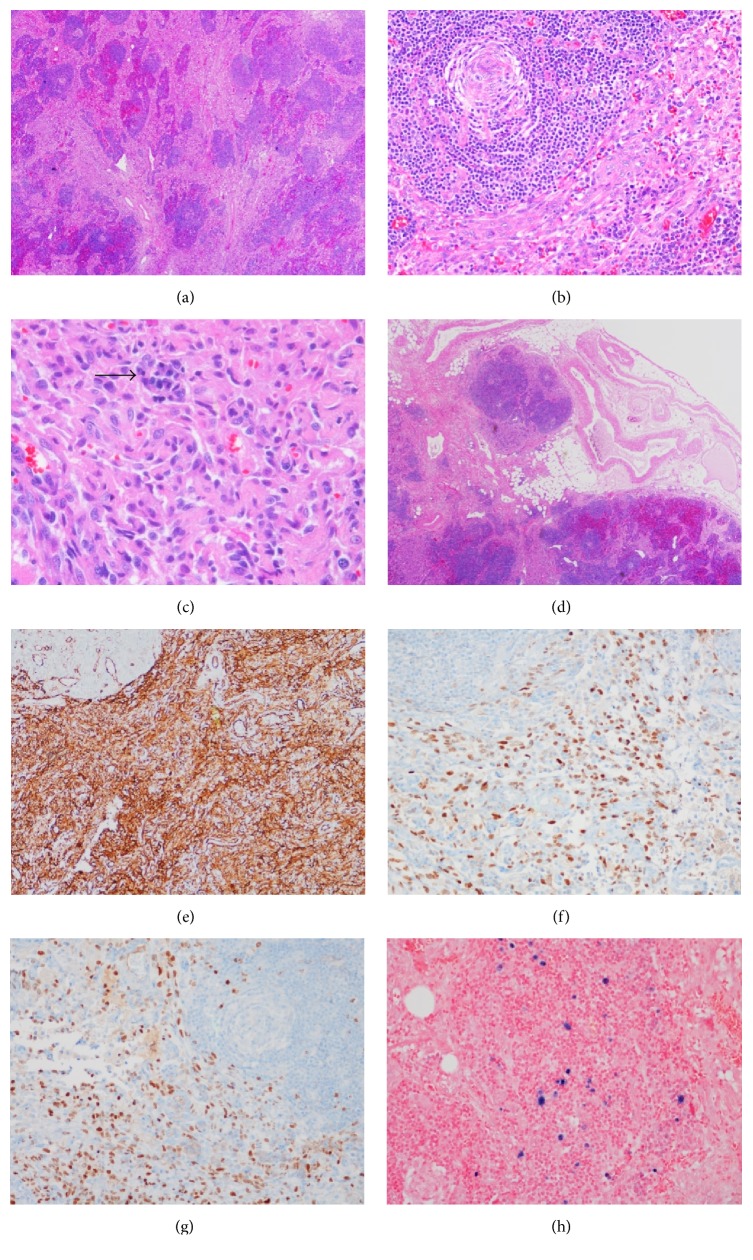
Section of the lymph node shows numerous atrophic germinal centers with marked vascular proliferation in the interfollicular area along with increased number of plasma cells (arrow); (a) ×40, (b) ×100, and (c) ×200; focal vascular malformation is noted near the capsule (d) (×40); Immunostains CD31 and ERG highlight vascular proliferation (e) and (f) (×200), respectively; a large portion of the vascular endothelial cells and scattered cells in lymphoid follicles are positive for herpes virus type 8 (HHV8) by immunohistochemical staining (g) (×200); focal increased number of Epstein-Barr virus (EBV) positive cells are also noted by in situ hybridization for Epstein-Barr virus-encoded RNA (EBER) (h) (×400).

A 59-year-old Caucasian male with a history of hepatitis C cirrhosis and history of hepatitis B exposure presented 8 months after orthotopic liver transplant (LT) with fever, fatigue, myalgia, night sweats, nonproductive cough, and shortness of breath. He received tacrolimus (TCR) 3 mg twice daily and prednisone 7.5 mg daily for immunosuppression. He was afebrile at the time of admission and vitals were stable. His physical exam was normal. White blood cell count was 2.9, hemoglobin 7.1, hematocrit 23, platelets 94,000, AST 44, ALT 64, total bilirubin 1.5, albumin 2.7, protein 6.4, and INR 1.5. The basic metabolic panel was normal. Infectious workup including blood culture, urine culture, CMV, HIV, hepatitis A/B/C serologies, and urine histoplasma antigen was negative. CT scan of the chest showed borderline enlarged axillary lymph nodes. CT scan of the abdomen/pelvis showed moderate retroperitoneal adenopathy and ill-defined soft tissue density within the region of the porta hepatis. Bone marrow biopsy was performed for pancytopenia and showed hypercellular bone marrow (80%) with erythroid hyperplasia and dyserythropoiesis. Flow cytometry showed polyclonal B-cells and plasma cells; no evidence of lymphoma was found. Epstein-Barr virus (EBV) DNA in bone marrow was positive. Periaortic lymph node biopsy showed human herpes virus 8 (HHV8) associated Castleman's disease and marked vascular proliferation (Figure 1). Immunostain for HHV8 was negative in the bone marrow. Quantitative HHV8 DNA was 611,000 DNA copies/mL. EBV antibody was positive; level was less than 2.70. The patient was started on valganciclovir 900 mg daily, prednisone dose was decreased to 5 mg daily and later discontinued, and rituximab was started. Tenofovir 300 mg daily was started for hepatitis B exposure with negative hepatitis B DNA and positive core antibody. He completed 8 doses of rituximab. Clinical improvement was noticed. TCR dose was reduced to 1 mg twice daily. His tacrolimus levels before dose reduction were between 6.6 and 8.3 ng/ml and after dose reduction they were between 4.6 and 6.5 ng/ml. He required filgrastim for neutropenia. Follow-up CT scan showed decrease in size of lymphadenopathy. HHV8 DNA level was <1000 DNA copies/mL after 4 months and undetectable at 6 months of treatment. Valganciclovir was discontinued after HHV8 viral load remained negative for 3 months. He did have recurrence of hepatitis C and was successfully treated with a combination of Sofosbuvir and Simeprevir. His HHV8 DNA level has remained undetectable.

## 3. Discussion

Castleman's disease is a rare complication after LT and is associated with HHV8 reactivation or new infection. PTLDs are a serious and often devastating malignant complication of solid organ transplantation (SOT) and Epstein-Barr virus (EBV) is a key pathogenic driver in many PTLD cases [2]. It is extremely rare for a coinfection with EBV and HHV8 and subsequent development of Castleman's disease and EBV associated PTLD. In immunosuppressed organ recipients, EBV and HHV8 coinfection can cause neoplastic proliferation or nonneoplastic infectious complications which can be life threatening. Since prevalence of HHV8 is low, patients undergoing transplant are not routinely screened for it. There are 5 cases of Castleman's disease after LT described in the literature. A pediatric patient was treated with tapering of immunosuppression and switching TCR to sirolimus. The graft function remained stable, and repeat imaging showed regression of the lymphadenopathy [3]. One patient was treated with valganciclovir and cessation of immunosuppression. PCR 3 months into the treatment was negative for HHV-8 and a follow-up CT scan showed a dramatic reduction of lymphadenopathy [4]. In one case, TCR dose was decreased but the patient died with disseminated Kaposi's sarcoma [5]. One patient died after the treatment [6]. Vijgen et al. described a case of a liver transplant patient who presented with acute febrile illness 1 year after transplant and was later on diagnosed with Kaposi's sarcoma, HHV-8-associated multicentric Castleman's disease with microlymphomas, and a severe hemophagocytic syndrome on autopsy [7]. Riva et al. [1] described possible treatments: rituximab, reduction of immunosuppression and/or switching from calcineurin inhibitor to sirolimus, using valganciclovir, and monitoring of HHV8 viral load. Since FDA has issued a black box warning against rituximab for possible reactivation of hepatitis B, patients should be screened for this disease. Since most of these patients can have a fatal outcome, Castleman's disease should be considered among the differential diagnosis in LT patients presenting with fever and new onset lymphadenopathy. We believe that this is the first reported case with a 3-year follow-up.
